# Harm reduction strategies for cannabis-related problems: a literature review and typology

**DOI:** 10.1007/s00406-024-01839-3

**Published:** 2024-06-27

**Authors:** Jonathan Pratschke

**Affiliations:** Department of Social Science, University of Naples Federico II, Vico Monte della Pietà, 1, Naples, 80138 Italy

**Keywords:** Cannabis, Harm reduction, Systematic review, Typology, Interventions

## Abstract

Measures that seek to minimise the health and social consequences of substance use are an integral part of national drug strategies in many European countries. Against the backdrop of a high prevalence of cannabis use in the economically advanced countries, and increasing demand for treatment for cannabis-related problems, a range of harm reduction measures have been implemented by peers, statutory bodies and third-sector organisations. Based on a systematic review of the literature, the author describes these different forms of intervention, identifies innovative strategies and presents a simple typology that can be used when exploring existing measures or seeking to develop new policies. This typology covers different kinds of legal, socio-organisational and health-related interventions. All study designs were eligible for inclusion, with the exception of case reports, non-systematic reviews, editorials and news stories. Studies had to be published between 2011 and 2022, in English, and they had to refer to Europe, the Americas, Australia or New Zealand. A two-concept search was implemented using Embase.com and a number of other databases, combined with citation searches and manual website searching to improve coverage of research reports and advocacy documents. A total of 35 documents were deemed eligible, many of which rely on qualitative research methods.

Measures that seek to minimise the health and social consequences of substance use are an integral part of national drug strategies in most European countries. There is considerable variation in the adoption of harm reduction measures due to differences in policy aims and evolving social and cultural trends at national and regional level [1]. These measures aim to reduce or eliminate drug-related consequences rather than reducing or eliminating substance use and can involve a combination of policies, interventions and individual-level strategies. Until recently, harm reduction interventions generally focused on opioids and related problems, with some policy-makers fearing that harm reduction programmes for cannabis could be interpreted as condoning or encouraging its use [2, pp. 692–3].

Against the backdrop of a high prevalence of cannabis use in the economically advanced countries, a range of measures have been implemented in recent years, including top-down interventions and grassroots initiatives. In this article we describe these interventions and present a typology of cannabis-related harm reduction measures. Our hope is that this overview can help policy-makers in European countries as they seek to reduce the harms associated with the production, distribution and use of this drug. Far-ranging debates about drugs policy are under way in many countries, and a robust evidence base is needed in order to put effective policies in place to safeguard the health of the population.

A number of systematic reviews have already been carried out which are pertinent to this topic [3–8]. Bahji and Stephenson [3] summarise the international literature on the implications of cannabis legalisation, and conclude that prevalence of use tends to increase following legalisation, leading to a rise in cardiovascular mortality but also a reduction in opioid prescribing. The rate of Accident and Emergency admissions for cannabis-related problems increased in US states which legalised recreational or medical use of this substance, while the number of arrests fell dramatically and a significant reduction was observed in relation to crimes like rape and theft.

Campeny et al. [4] carried out a review of reviews in an attempt to identify cannabis-related harms. As far as mental health is concerned, the included studies document a risk of psychosis, affective disorders, anxiety disorders, and dependence associated with cannabis use. The main organic risks include respiratory difficulties, impaired cognition, gastrointestinal effects and disturbances involving the nervous system. Other consequences include traffic accidents and social harms due to being arrested or stigmatised. The authors note that there is still no consensus in the literature on what constitutes heavy use, and how the aforementioned risks may vary by type of cannabis, quantity, frequency and duration of use.

Fischer et al. [5] identify a series of evidence-based lower-risk cannabis use guidelines, some of which involve prevention rather than harm reduction. In terms of harm reduction, the authors mention measures like promoting alternative methods of consumption, not driving while under the influence and using products with a similar quantity of THC and CBD. González-Ponce et al. [6] explore harm reduction strategies by reviewing research on university students who use both alcohol and cannabis. Their main finding is that individual-level harm reduction strategies such as limiting the amount of cannabis smoked in one sitting or only using cannabis with trusted peers could be helpful to students with poor mental health, high impulsivity, and low self-regulation.

Razaghizad et al. [7] conclude that there is some evidence to support the effectiveness of interventions that seek to reduce driving under the influence of cannabis; warnings on cannabis packaging can increase awareness of this risk. For adolescents and young adults, there is also evidence that motivational interviewing can reduce the risks associated with driving under the influence. Richards et al. [8] analyse published reports of unintentional cannabis ingestion among children and conclude that this is a serious public health issue which clinicians need to be aware of. They note that many commercial cannabis-infused edibles on sale in the U.S. look like biscuits, sweets, chocolate or cakes. Their findings lead them to propose better public health campaigns to reduce the risk of exposure in children, to limit home production, and to improve warning labels and wrappers.

The aforementioned literature reviews mention some useful harm reduction measures but do not provide a comprehensive treatment of this important issue. This study seeks to fill this gap by providing an up-to-date and wide-ranging review of the harm reduction measures for cannabis-related problems that have been identified in the literature. No attempt is made to evaluate their effectiveness, which is not possible given the current state of research, and would require a different kind of research. This paper is based on an evidence review (which is a specific type of systematic literature review) which includes different types of publication – grey literature as well as book chapters and journal articles – using qualitative and observational research methods as well as randomised controlled trials.

## Search strategy

Our research questions do not fit within a standard PICO structure so we have used our own format, including population, setting, focal issues and other eligibility criteria (Table 1). Eligible publications were not assessed in relation to design or methodology as we are not concerned with measuring impacts. Interventions that seek to reduce harms by avoiding or reducing cannabis use are not included, as these fall under the heading of prevention.

**Table 1:** Eligibility criteria for systematic review

Population	*People who produce, sell or use cannabis*
Setting	*Harm reduction interventions*
Focal issues	*Good practices; innovative measures*
Study designs	*All research designs with the exception of case reports on single individuals, non-systematic reviews, commentaries, editorials and news stories*
Other limits	*Studies in English, studies published between 2011 and 2021 Europe, the Americas, Australia and New Zealand*

We executed our main search using Embase.com, as this is a large health research database with around 37 million records (including MEDLINE). Embase.com offers a sophisticated search interface with proximity operators and tools for handling synonyms, which improves the efficiency of searches. We adopted a two-concept approach combining ‘cannabis’ and ‘harm reduction’. There are a range of Emtree terms available, including ‘cannabis’ (41,345 hits) which covers various synonyms and was judged to provide satisfactory coverage of our first concept. The Emtree term ‘harm reduction’ (7,754 hits) is also useful, although it is necessary to add two further elements in order to achieve a satisfactory coverage, due to reliance on different terms in the literature.

The first of these elements relates to the concept of harm, and includes the terms harm*, risk*, low-risk, lower-risk, danger*, damag*, impair*, hazard*, effect*, expos*, impact*, conseq*, implicat*. This enables us to identify records which use these terms or negations/extensions of them in their title or abstract (such as less harmful, less risky etc.). The second element relates to the concept of reduction, and includes terms like reduc*, avoid*, lower, prevent*, curtail*, moderat*, mitigat*, eliminat*, minimis*, minimiz*, manag*. These two elements are then combined using proximity operators and applied in parallel to the main Emtree search terms in order to improve our coverage of the literature (Table 2).

**Table 2:** Search algorithm for Embase.com

Set number	Search query	Records identified
#8	#7 AND [abstracts]/lim	650
#7	#5 AND #6	744
#6	2011:py OR 2012:py OR 2013:py OR 2014:py OR 2015:py OR 2016:py OR 2017:py OR 2018:py OR 2019:py OR 2020:py OR 2021:py OR 2022:py	17,537,718
#5	#1 AND #4	952
#4	#2 OR #3	93,468
#3	((harm* OR risk* OR ‘low-risk’ OR ‘lower-risk’ OR danger* OR damag* OR impair* OR hazard* OR effect* OR expos* OR impact* OR conseq* OR implicat*) NEAR/2 (reduc* OR avoid* OR lower OR prevent* OR curtail* OR moderat* OR mitigat* OR eliminat* OR minimis* OR minimiz* OR manag*)):ti, kw	86,178
#2	(‘harm reduction’):exp, ti, ab, kw	11,459
#1	(cannabis): exp, ti, ab, kw	61,231

As well as searching Embase.com, we interrogated other databases and carried out a purposive web search, including databases such as the CENTRAL Register of Controlled Trials, the Cochrane Database of Systematic Reviews, Epistemonikos and the British Library catalogue. A number of other websites were searched, based on expert knowledge, leading to the identification of 94 potentially relevant documents, in addition to the 650 we found using Embase.com.

The records identified using this search strategy were loaded into Zotero for rapid screening. The aim of this process was to exclude publications which were obviously ineligible as a result of their study design, subject area (biology, chemistry, veterinary science) or other characteristic. This led to the exclusion of 158 records. The remaining documents were merged with the results of the purposive web search and de-duplicated, leaving 554 records to be screened on title and abstract. We screened each record using information from the title and abstract fields to identify potentially eligible documents, leading to the identification of 89 records^1^.

**Fig. 1:**
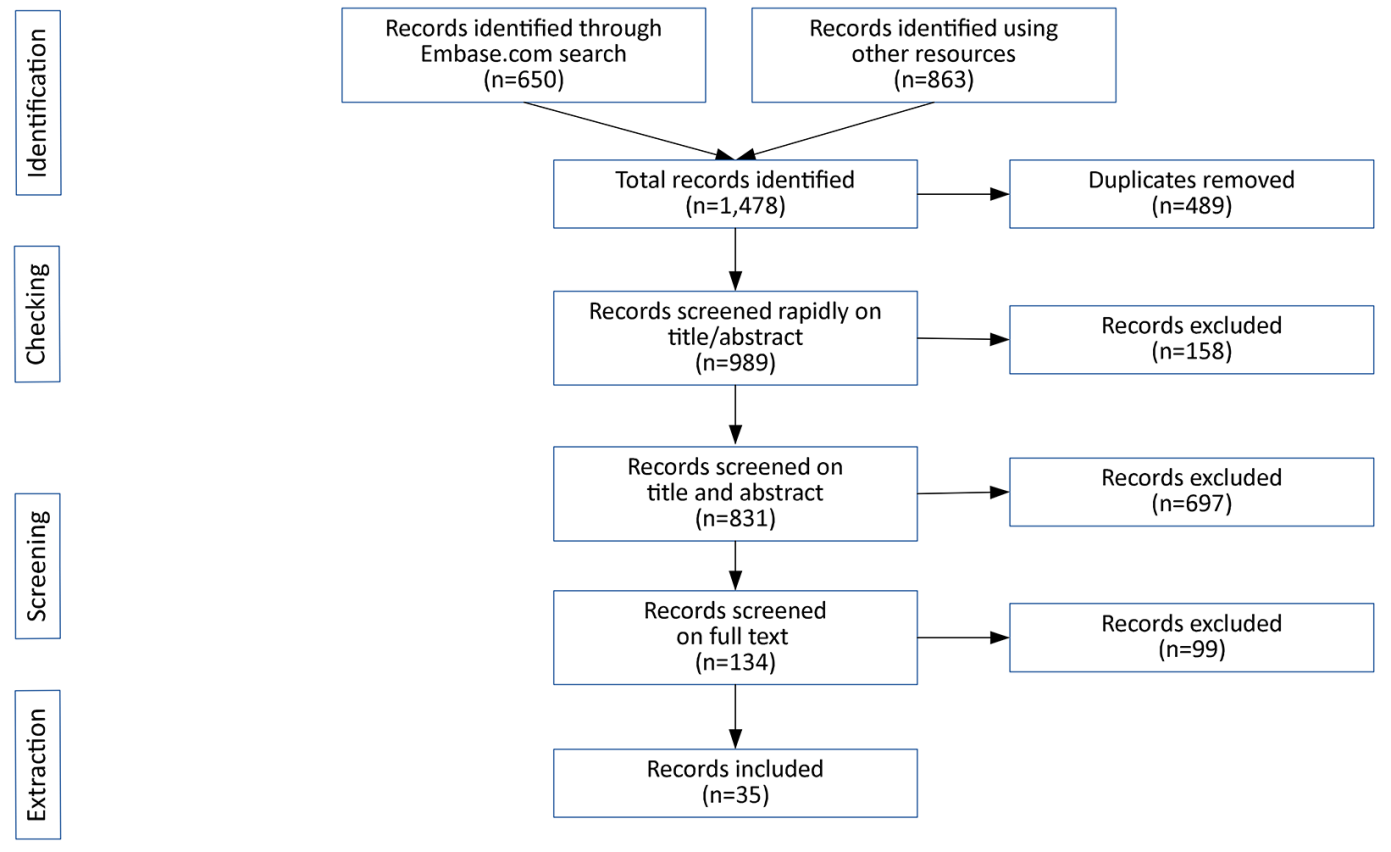
PRISMA flow diagram for systematic literature review

Documents were considered eligible for inclusion in the literature review if they discussed interventions, programmes, projects or initiatives that aim to reduce harms associated with the production, distribution or consumption of cannabis. The full text of each potentially eligible document was acquired and we assessed the eligibility of each document, leading to the identification of 25 records. As well as inspecting the publications referenced in the papers themselves, we used Harzing’s *Publish or Perish* software [9] to identify records which cite these publications. This is an effective way to identify recent publications which may not yet have been indexed. After removing records which overlapped with our earlier Embase search, we identified 268 new records; 35 of these were selected for full text screening [1, 2, 10–42]. A number of additional publications (9 in total) were identified during the process of full-text screening, and after screening all of these records an additional 10 eligible documents were identified. Figure 1 summarises the search process using a PRISMA diagram.

We carried out a narrative synthesis of our final set of 35 eligible studies. We used a data extraction sheet to gather together summary information for each publication, including the location, context, type of intervention, study design, and findings. After carefully reading each publication, we identified a number of themes. We continued compiling and organising the evidence until we had achieved a satisfactory degree of saturation and assigned all relevant findings to a theme. Refinement and selective merging of themes enabled us to develop a typology of harm reduction initiatives in the context of cannabis production and use.

## Typology

Some attempts have already been made to construct typologies of harm reduction interventions for cannabis. Hall and Degenhardt [2] distinguish between (1) advice to cannabis users about how to reduce risks; (2) specific interventions for problem cannabis users; (3) policies that rely on criminal sanctions to deter people from using cannabis in harmful ways; (4) legislative approaches that aim to reduce the harms of cannabis prohibition (p. 693). MacCoun, Reuter & Schelling [43] distinguish between health, social and legal risks when discussing drug-related harms. These approaches illustrate how typologies can focus on either the form of intervention or type of harm. It is possible to combine these approaches, although this leads to redundant categories. The final version of our typology distinguishes between three broad strategies of intervention (legal, social and health-related) and identifies three types of measure within each. This yields nine categories which cover the main forms of harm reduction that have been discussed in the literature.

## Legal interventions

### Legalisation, decriminalisation and toleration of cannabis

The prohibition and stigmatisation of cannabis use, and resulting legal and social problems, are considered by many researchers as important sources of harm [25]. Conversely, the removal of criminal penalties not only reduces the risk of arrest, but also opens up a space for innovative policies and strategies. Before the legalisation of cannabis for adult recreational use in Canada in 2018, Uruguay was the only country that had legalised possession and use at national level [12]. The law in Uruguay indicates three ways of legally obtaining cannabis: domestic cultivation, cannabis clubs, and pharmacies; sharing cannabis is also legal. There has been an increase in the use of cannabis among adults and adolescents in Uruguay since legalisation, with adult prevalence rates (for use over past year) increasing from 9% in 2014 to 15% in 2017. The user base has widened to include a greater percentage of women and older users [12]. Cannabis sold in pharmacies is organic, and tests carried out on homegrown cannabis show that this does not generally contain toxic substances such as contaminants, fungi, and additives, which are often present in cannabis imported illegally into the country. Legalisation is considered to have brought legal, social and health benefits [12].

Although cannabis legalisation has been expanding in the USA in recent years, European countries have not yet moved decisively in this direction [13]. One reason for this is that they are signatories to United Nations drug control conventions which oblige them to criminalise cannabis production and to treat possession in non-medical contexts as a punishable offence. Nevertheless, several countries have adopted forms of de jure or de facto decriminalisation.

### Product, labelling and packaging regulations

One of the advantages of regulated markets for cannabis is that they offer the possibility of reducing harm by ensuring that available products are of high quality and helping consumers to use them in appropriate ways. Researchers often argue that legalisation is more effective than decriminalisation from a harm reduction perspective. Regulation poses challenges in this context due to the existence of different types of product, outlet and modes of use. The popularity of alternative cannabis products has increased rapidly in countries and states where they can be purchased legally [23, 34]. However, this increases the risk that cannabis products may be consumed unintentionally by children, pets, or unsuspecting adults.

In Colorado there are no restrictions on the types of edible cannabis products that can be sold, which can include sweets, chocolates and any kind of snack [39]. Nevertheless, labels on cannabis products must be conspicuously marked with a standardised cannabis symbol and edibles must have a date showing when the food was made and a complete list of ingredients [34]. In Washington State, regulations prohibit any statement, depiction, or illustration showing a child or other minor consuming a cannabis edible product [38]. Each product must include a list of ingredients, major food allergens, and a delayed effects warning. Nevada’s labeling provisions resemble those of Washington State, with all products requiring a bold label. Packaging must not include images that represent a cartoon character, mascot, action figure, balloon or toy or anything else that could appeal to children [38]. Nevada prohibits edibles from bearing any likeness to lollipops, ice creams, real or fictional characters, animals, fruit or practically any commercially available candy or snack. All edible products must have a conspicuous and clear label containing a range of information and warnings.

The availability of legal cannabis in new forms and formats has significantly altered the culture of cannabis consumption. Small and convenient vaporiser pens have minimal smell and are attractive to segments of the population which may not be accustomed to smoking cannabis flowers in more traditional ways. Vaporiser pens are perceived as being more fashionable, discrete, easier to use, less harmful and less stigmatising than other forms of consumption [34]. However, all aspects of these devices require testing and regulation to reduce risks such as inhaling harmful substances.

In all legalised cannabis markets, the percentage or weight of THC and CBD in products is clearly labelled, which is a considerable advantage. What is missing is often a clear expression of drug dose in terms of amount of product [34]. Labelling regulations based on the principle of dose expression would indicate the amount of a product that corresponds to a standard THC serving, rather than relying on consumers to make their own mathematical calculations.

### Preventing driving under the influence of cannabis

Epidemiological studies show that cannabis users who drive while intoxicated are at increased risk of motor vehicle crashes [2]. The percentage of adolescents driving after using cannabis or riding with a driver who has used cannabis has increased in the USA over the past two decades, and now surpasses the alcohol-related driving and riding rates. Knight et al. [24] carried out a baseline and follow-up study to assess whether a brief intervention to reduce driving/riding risks might be effective in a sample of adolescents. After adjusting for baseline riding and other covariates, those who received counselling had a 30% lower risk of riding with a drinking driver and 54% lower risk of riding with a driver who had used cannabis at the 3-month follow-up, although these effects had dissipated after about 12 months.

To reduce the number of road traffic accidents, several countries have introduced random roadside testing and drivers can be tested for cannabis if they are involved in an accident, are observed to be driving erratically or suspected of being intoxicated. Biochemical measures of cannabis in blood, urine or saliva are sometimes used in addition to psychomotor assessments when considering the level of impairment of a driver and deciding whether to prosecute. Establishing legal limits for driving under the influence of cannabis is difficult [41]. There are two broad approaches: the ‘impairment approach’ (where a driver can only be prosecuted if their driving is actually impaired) and the per se approach (where a driver can be prosecuted if the level of THC in their body fluids exceeds a defined threshold). Sixteen countries in Europe have legal non-zero THC concentrations at or above which drivers are automatically prosecuted, with 11 of these setting specific thresholds.

To recapitulate, in jurisdictions where cannabis is legal, risks are often addressed through regulations regarding packaging and labels. Researchers stress the need to provide users with a clear indication of how much of a given product they should take if they want to consume a ‘standard dose’. As Thompson [38, pp. 70–1] observes, the inadvertent ingestion of cannabis edibles by children poses a great threat to society, and the reduction of this risk requires a combination of measures. From the perspective of driving, systems which include biochemical measures of intoxication appear to have the potential to reduce traffic accidents.

## Socio-organisational interventions

### Cannabis social clubs

Since the 1990s, cannabis users in Europe have developed proposals to reduce legal insecurity and to improve access to high-quality products [15]. For example, collective cannabis plantations and Cannabis Social Clubs (CSCs) were introduced by activists in Spain as an alternative to the black market. CSC’s are non-profit associations whose members are adult cannabis users. People who join a club have to satisfy certain conditions and together organise the professional cultivation of cannabis to cover their personal needs, against the backdrop of security and quality checks [11].

The CSC model has been emulated in Belgium and Uruguay as well as other countries. There is evidence of experimentation with this model in Chile, Colombia, Argentina, the United Kingdom, France, Slovenia, Switzerland, New Zealand, and Italy [18]. Researchers have argued that CSCs minimise the risks to cannabis users by helping them to avoid legal problems, by providing a high-quality product, protecting minors, making information available, and reducing the stigma associated with cannabis use [14, 29].

Obradors-Pineda et al. (2021) examine the harm reduction practices introduced by 15 CSCs in Catalonia. Most of these had between 50 and 200 members and half had a member of staff who had received training on cannabis risk reduction. Decorte [18, p. 123] describes CSCs as occupying a meaningful middle ground between cannabis prohibition and commercial legalisation. The European Coalition for Just and Effective Drug Policies (ENCOD) promotes the CSC model and has produced a set of guidelines that highlight the ability of CSCs to provide information and advice, facilitate interactions between members, organise workshops and talks, promote alternatives to smoking and refer members to treatment services [21, p. 32].

Several risks have been identified in relation to CSCs, starting with the possibility that they can become opportunistic, profit-seeking organisations [18, 19]. There is a risk of displacing chaotic users into non-controlled environments, while excessive regulation of CSCs could curb users’ willingness to take part in them [15].

### Promotion of self-cultivation and social supply

One way to deal with a lack of legal access to cannabis is to turn to home-growing or to rely on home-grown cannabis supplied by others. In overall terms, there has been a shift in recent years from imported cannabis to domestic production, involving both small-scale and large-scale growers [31, 35]. The trend towards domestic production utilising more technically advanced methods began in earnest in the 1990s and accelerated in the early 2000s due to easier access to information and equipment. Many growers regulate the potency of their cannabis, are proud of the quality of their plants and cultivate cannabis to avoid having to pay drug dealers for poor quality products [35]. One benefit of this model is that it reduces or eliminates the risks associated with large plantations, including the exploitation of workers who are forced to operate in dangerous environments.

Interest in using cannabis to manage the symptoms of medical conditions has also increased in recent years, and one survey found that 24% of growers in Denmark and 59% of those in Finland were producing cannabis for medical purposes [22]. The majority of medical growers in Australia, Belgium, Denmark, Finland, Germany and the UK have a medical diagnosis and approximately one in five report that their doctor suggested using cannabis. Potter & Klein [35] report that up to a million people in the UK could be using cannabis (illegally) for medical purposes, with a substantial number growing their own plants.

In Belgium, small-scale cultivation for personal use is tolerated, and legislative reform in 2003–2004 made the possession of 3 g of cannabis and the cultivation of one female plant for personal use tolerable (albeit still criminal) offences. The aim was to separate cannabis from other drugs and to grant the prosecution service more discretion when tackling cannabis-related offences [31]. In 2004, Western Australia became the fourth Australian state to implement a civil penalties scheme for minor cannabis offences. Those apprehended with small amounts of the drug could, depending on the circumstances, be eligible for a fine or infringement notice instead of being arrested. The scheme also allowed adults to grow two cannabis plants outdoors, or to possess up to 30 g of dried cannabis flowers without risking arrest. There was no evidence of an increase in cannabis use following introduction of the scheme and no evidence of an increase in the amount of cannabis being cultivated [26], although this policy was later reversed after a change in government [44].

Home cultivation capitalises on the spontaneous health-promoting behaviour of non-commercial growers, many of whom are embedded in a cannabis culture that values the natural qualities of this drug [13]. At the same time, unregulated home cultivation could make it easier for minors to access cannabis and reduce the ability of the state to collect taxes and to regulate access and pricing.

### Cannabis dispensaries and coffee-shops

Researchers have drawn attention to the positive features of systems for the distribution of cannabis which involve a combination of national regulation and local control. An example is the Dutch coffee-shop model, which was made possible by the decision to not enforce criminal sanctions for the sale and possession of small quantities of cannabis. The objective of Dutch cannabis policy is to limit risks for cannabis users and to reduce harms to the wider society. In particular, it aims to prevent cannabis users from becoming marginalised, stigmatised and criminalised, and to reduce access to more harmful substances by separating the supply lines of these substances.

Coffee-shops are café-like places where the sale of cannabis is tolerated as long as there is no advertising, no hard drugs, no nuisance to other people, no access for minors and as long as no more than 5 g of cannabis is purchased [27]. Although their number is gradually decreasing, coffee shops are still a widespread phenomenon in the Netherlands and it is estimated that in municipalities with officially tolerated coffee-shops, around 70% of cannabis consumed is purchased from these outlets. The coffee-shop system appears to be successful in separating the hard and soft drug markets; where there is a lower density of coffee-shops, there are more illicit dealers [27]. Survey data show that both last year and last month prevalence rates in the Netherlands have remained stable in recent years and are well below the European average.

The production and sale of cannabis in wholesale quantities remains illegal in the Netherlands, which makes it difficult for coffee-shops to exert control over the quality of the product they sell. Cannabis samples from Dutch coffee-shops have at times been found to be contaminated with mould, bacteria, pesticides, heavy metals or additives. Moreover, coffee-shops can encourage cannabis tourism, an issue which is increasingly attracting the attention of policy-makers [27].

Dispensaries emerged in Canada prior to the legalisation of medical cannabis, emulating the local dispensaries that were established following the 1996 medical cannabis ballot in California. Their purpose was to provide high-quality cannabis to patients with a medical condition for which cannabis had been recommended by a licenced health care provider. For older adults, in particular, dispensaries represented an accessible and non-stigmatising source of medical cannabis. The dispensaries formed an association and adopted a form of self-regulation, like CSCs in Europe.

The study by Lau et al. [25] provides insights into the potential role of dispensaries in terms of harm reduction. They interviewed almost 100 older adults living in San Francisco who were born between 1946 and 1964 and had used cannabis a minimum of 24 times over the past six months. Those who had access to medical cannabis dispensaries were found to be more knowledgeable about alternative delivery systems and the use of edibles. The people Lau et al. interviewed were engaged in sensible, continuing, controlled use which was perceived as relatively unproblematic by the individuals concerned.

People working in cannabis dispensaries often provide information to consumers and have been identified as an important resource in delivering harm reduction initiatives and advice [17]. There are roughly 4,000 budtenders in Washington State alone, and Carlini et al. [17] studied their orientations and practices using focus groups. Both medical and non-medical customers purchase cannabis in the same stores, and budtenders with certified medical consultant training can serve both kinds of customer. When asked about how they help people, budtenders talked about helping clients to avoid the risk of acute cannabis intoxication and to understand the need for safe cannabis storage.

## Health-related interventions

### Service provision, training and advice

Medical cannabis is legal in a number of countries, although supply is organised in different ways. In integrated systems, cannabis products are made available through pharmacies or delivered directly to the patient by a licenced distributor. In addition to these official sources of supply, patients may obtain cannabis from dispensaries, coffee-shops, compassion clubs or CSCs, or they may grow their own, as we have seen.

The document written by Nathoo et al. [28] is an evidence-based harm reduction resource on breastfeeding and cannabis which is targeted at health and social care providers in Vancouver. It aims to influence professionals by improving their knowledge and awareness. The authors encourage care providers to create a space for discussion with breastfeeding mothers, urging them to be trustworthy and respectful during interactions. They describe a range of strategies for harm reduction, such as using cannabis with a lower concentration of THC, avoiding synthetic cannabis products and using cannabis that is homegrown or purchased from a licensed retailer. Nathoo et al. [28] suggest that mothers should try not to breastfeed immediately after using cannabis, and take steps to avoid breathing second-hand cannabis smoke and exposing their babies to it. There is a section on parenting strategies where the authors underline the different ways in which cannabis can affect a parent’s ability to pay attention to their baby, to recognise dangers, and to make decisions in an emergency. They sugges that mothers who use cannabis need to plan their parenting activities to reduce harm to young children. There is scope for integrating this kind of material within training programmes for health and social care professionals.

### Promotion of less harmful modes of use

A number of harm reduction techniques relating to mode of use have been discussed in the literature. When smoking cannabis, it is advised to refrain from using tobacco, for example, and to use cannabis that is free of contaminants and adulterants, avoiding prolonged breath holding and to use alternative delivery systems such as pipes or vaporisers [1, 25]. Other authors have emphasised the value of adopting alternative methods of consumption, such as edibles [38].

Alternatives to smoking cannabis – such as oral administration and vaporisers – have attracted a considerable amount of attention in this literature. The evidence suggests that vaporising as an alternative to smoking can produce improvements in respiratory function, such as reduced coughing, wheezing, shortness of breath, tightness of chest and phlegm [25]. Encouraging cannabis users to switch to vaporisers is thus a potentially useful harm reduction measure. As vaporisers are a commercial product, with specific advantages and drawbacks, the decision about whether to purchase one is typically left to the individual.

### Cannabis testing and quality control

Drug testing services have been a valuable component of drug-related harm reduction strategies for many years. These services provide the results of a chemical analysis of substances that are submitted by the public, or seized by the police, as well as engaging directly with people who use drugs. The value of testing cannabis products is highlighted by Oomen et al. [30], who describe how testing services in one case found evidence of adulteration of cannabis products with a synthetic cannabinoid receptor agonist. They suggest that lack of familiarity with drug testing services might prevent cannabis consumers from coming forward and having samples analysed. Particularly in the European context, where cannabis growers, CSCs and coffee-shops often do not have the resources to test their own products, and where underground markets retain a dominant position, enabling individual citizens to submit samples for testing is a potentially useful harm reduction measure.

Following the legalisation of recreational cannabis in Canada, the federal government developed a strict regulatory framework for distribution which included quality assurance protocols [36]. Health Canada is in charge of overseeing cannabis production; licenced producers are required to have protocols in place to ensure that their products are safe. Licenced cannabis producers can only use a small number of natural pesticides with a low risk of toxicity [36]. Other measures include checking for microbial contaminants, pesticide residues, elemental impurities and residual solvents. Seltenrich [37] discusses regulations in US states regarding the use of pesticides during cannabis cultivation, highlighting the following paradox: because cannabis is illegal under federal law, no pesticides have been approved by the EPA. As a result, each state that has legalised cannabis has adopted different rules regarding tolerated pesticides and related regulations.

## Conclusions

Harm reduction measures for cannabis have been developed in piecemeal fashion in a number of countries. Building on this experience, it is now possible to adopt a more systematic approach to the promotion of health and well-being among cannabis users. The reduction of risk often relies on regulations, protocols and the provision of advice by professionals, which is why most of the research summarised in this article comes from states and countries where medical or recreational cannabis use is tolerated or legal. There are many different ways of regulating access to cannabis, however, and researchers underline the importance of integrating an awareness of cannabis-related harms and harm reduction measures into high-level policy debates. The typology of harm reduction measures presented in this article is a potentially useful tool when seeking to develop an evidence-based framework for reducing drug-related harms. Harm reduction measures can be implemented at both the individual and the aggregate level, via legal norms, social innovations and the provision of information and services. The measures described in this article have not generally been subjected to rigorous evaluation, and this issue requires urgent attention. In the context of widespread changes in policy and a shift towards paradigms which are centred on health promotion, an evidence base for harm-reduction measures in the context of cannabis-related harms would be extremely useful. This will require new research on specific interventions using appropriate research designs, with a view to providing policy-makers with better guidance on harms and harm reduction measures in the future.

## Footnotes

1 At this stage of the search we included documents which addressed harm reduction from a research perspective as well as those which discussed programmes and interventions. This enabled us to identify a larger range of evidence-based practice guidelines and recommendations, which are reported separately.
